# Proton Pump Inhibitors and Kidney Disease: What Gives?

**DOI:** 10.34067/KID.0000000000000521

**Published:** 2024-07-19

**Authors:** Mark A. Perazella

**Affiliations:** Section of Nephrology, Yale School of Medicine, New Haven, Connecticut

**Keywords:** CKD, drug nephrotoxicity

The proton pump inhibitors (PPIs) are a class of medication commonly dispensed by prescription and frequently acquired by over-the-counter purchase in some countries. They are used for a variety of gastrointestinal illnesses ranging from acid indigestion and gastric dyspepsia to gastro-esophageal reflux disease and premalignant esophageal disorders such as Barrett esophagus. As such, they are widely consumed in many countries around the world. The beneficial gastrointestinal effects gained by many are not without a number of adverse consequences some that include various infections, magnesium loss and other nutritional deficiencies, bone fractures, and kidney injury. As a nephrologist, I will address the issue of kidney injury in this perspective.

In 2016, observational studies reported an association of PPI use with development of CKD.^1^ I speculated at the time that CKD in this setting may have been due to unrecognized and untreated PPI-associated acute tubulointerstitial nephritis (ATIN) leading to chronic tubulointerstitial injury and fibrosis.^2^ Numerous observational studies and systemic reviews/meta-analyses generally confirming this association have subsequently been published.^3^ Although this is interesting food for thought, it by no means demonstrates a causal link. Furthermore, outside of the ATIN to CKD hypothesis, the PPIs have no clear-cut nephrotoxic mechanism to explain how these drugs cause CKD. In fact, two prospective multicenter open-label parallel group studies comparing the efficacy and safety of two PPIs with antireflux surgery for chronic gastro-esophageal reflux disease showed no major safety concerns in 298 patients with 5 years of follow-up and 298 patients with 12 years of follow-up, respectively.^4^ However, the pot has been stirred once again by the recent publication by Pyne *et al.* of a *post hoc* analysis of the Cardiovascular Outcomes for People Using Anticoagulation Strategies (COMPASS) clinical trial.^5^

Using the *post hoc* analysis of the COMPASS trial, the authors provide further observational evidence to support the contention that PPIs are associated with CKD, which was defined by a faster decline in eGFR compared with placebo. The COMPASS trial published in 2019 randomized patients with peripheral vascular disease or coronary artery disease to rivaroxaban and aspirin, rivaroxaban, or aspirin.^6^ PPI administration was randomized among participants not taking the drug at trial enrollment. Notably, among 17,598 participants randomized in the original trial, the authors did not observe an increase in any kidney outcomes including AKI, acute nephritis, nephrotic syndrome, or incident CKD. However, incident CKD may have been missed by the study methodology used to capture CKD complications. By contrast, the *post hoc* analysis by Pyne *et al.* examined change in eGFR collected within the year prior to baseline and in the open-label extension of 8991 patients that had follow-up testing. While the two groups had similar starting mean eGFRs (75 ml/min per 1.73 m^2^), the placebo group had a rate of decline of 21.41 ml/min per 1.73 m^2^ per year and the pantoprazole group had a rate of decline of 21.64 ml/min per 1.73 m^2^ per year. In adjusted analysis, a statistically significant faster eGFR decline of 0.27 ml/min per 1.73 m^2^ per year was noted for the pantoprazole group, a 0.89 ml/min per 1.73 m^2^ difference over a mean of 3.3 years. A nonstatistically significant trend was observed in the composite renal end point (eGFR <60 ml/min per 1.73 m^2^, case report form noted CKD, death from kidney failure, or follow-up exclusion because of eGFR <15 ml/min per 1.73 m^2^) in patients with an eGFR ≥60 ml/min per 1.73 m^2^ that were treated with pantoprazole. In addition, another negative result of this *post hoc* analysis was the observation that the rate of AKI did not differ significantly in placebo and pantoprazole-treated patients. This may once again be related to the data collection method used in this trial as AKI was ascertained using administrative codes rather than changes in serum creatinine, which would likely not detect subclinical episodes of AKI or ATIN.

How should we view these new *post hoc* data on the background of a plethora of positive observational data and systematic reviews/meta-analyses that support an association of PPIs with CKD? On the one hand, the results of this *post hoc* analysis lend credence to the numerous previously published observational studies that link PPI exposure to a slight increase in risk of CKD. On the other hand, the supportive data in this analysis are not earth shaking as the effect on eGFR by PPIs is small when viewed in clinical terms, the composite renal end point was not statistically different, and AKI was not an observed complication recognizing the data collection problem. As we digest these data, it is incumbent on us to review the strengths and limitations of the current study by Pyne *et al*.^5^ The strengths of the COMPASS trial and the *post hoc* analysis is the prospective, randomized nature of the trial. In particular, the PPIs were randomly allocated, which reduced the possibility of confounding by indication. In addition, a large number of patients were included in the study. Thus, this would make this trial a step up from many of the observational studies. One might conjecture that this should seal the fate of PPIs as a cause of CKD. What about study limitations? There are several to consider including the relatively short follow-up, although this may be a strength as longer follow-up may have shown a more impressive eGFR decline with PPIs. The use of clinically obtained eGFRs is clearly a weakness. It is recognized that there is imprecision with eGFR, and the small difference noted in the study can be considered in the margin of error. In addition, small changes in eGFR as in this analysis may occur due to changes in creatinine production (muscle mass, medications, *etc.*), impaired tubular creatinine secretion, or transient changes in glomerular filtration (volume depletion, RAAS antagonists, *etc.*). Thus, measured GFR would instill more confidence. Finally, the potential for selection bias exists with the requirement for enrollment in the open-label extension.

Another perplexing issue in the PPI-CKD story is the absence of a definitive mechanism of nephrotoxicity. PPIs are metabolized rapidly in the liver by cytochrome P450 enzymes, primarily cytochrome P450-2C19 and -3A4 and excreted by the kidneys with no effect on the renal H^+^K^+^-ATPase.^7^ These drugs cause gastrointestinal magnesium wasting based on their effects to reduce the intestinal transport of magnesium by the transient receptor potential-M6/7 channels by enterocytes.^8^ It has been theorized that hypomagnesemia may promote kidney dysfunction or enhance worsening kidney function through a number of processes.^9^ In addition, alterations to the gut microbiome are postulated as a potential mechanism of CKD.^3^ However, none of these is proven and they are far from accepted explanations. As I have put forward, it is possible that PPI-associated CKD develops from undiagnosed and therefore untreated ATIN.^3^ Why? First, ATIN is tricky to diagnose in the absence of high suspicion because it can occur as acute kidney disease with minimal serum creatinine changes that do not reach AKI levels in the Kidney Disease Improving Global Outcomes time frames. In addition, these patients often do not undergo kidney biopsy, which is the current gold standard as clinical and laboratory parameters have poor positive and negative predictive values.^10^ Undiagnosed and untreated ATIN can lead to chronic tubulointerstitial injury and fibrosis over time. Even ATIN diagnosed and treated may progress to CKD, especially when treatment is delayed more than 2–4 weeks.^10^ However, none of these potential mechanisms to explain PPI-associated CKD have been proven.

Thus, I think we are left with the following conclusion (recognizing this is far from a concluded issue): PPI exposure appears to be associated with an albeit slight increase risk of CKD. However, at the present time and in the absence of detailed data, the nature of this CKD risk remains unknown and unexplained. We clearly need more definitive data; however, we are unlikely to get it. So, what should we recommend to our colleagues and our patients? In general, the vast majority of patients are likely in no danger from kidney injury or disease from the PPIs. My own father has been taking them for nearly 30 years for heartburn and indigestion without any adverse effects despite my advice to stop them and see how he feels. As clinicians and health care providers, we must review the benefits and risks of a medication and combinations of medications for our patients. Encouraging our GI colleagues to prescribe the PPIs in a judicious manner (short courses) and for accepted indications is paramount. In addition, we should try to convince our patients to stop the drug when there is not a clear-cut indication regardless of stability of kidney function. Based on the widespread use of the PPIs, this will be an uphill battle—and we are not armed with definitive data to argue with.
